# Novel renoprotection methods by local and remote conditioning

**DOI:** 10.12861/jrip.2014.12

**Published:** 2013-11-03

**Authors:** Mehri Kadkhodaee, Zahra Sedaghat

**Affiliations:** ^1^Department of Physiology, School of Medicine, Tehran University of Medical Sciences, Tehran, Iran; ^2^Department of Physiology and Pharmacology, School of Medicine, Bushehr University of Medical Sciences, Bushehr, Iran

**Keywords:** Ischemia-reperfusion, Ischemic conditioning, Renoprotection

**Implication for health policy/practice/research/medical education:**

The possibility to create resistance against ischemic injury through ‘organ conditioning’ is an area of increasing interest. The remote ischemic conditioning may have an immense effect on the renal patients and in clinical practice in the near future. Remote ischemic per-conditioning is perhaps the most promising protective strategy among the adaptive surgical techniques.

By far, no integral treatment is available to improve outcomes in patients encountering renal ischemia-reperfusion injury (IRI), when blood supply to the kidney is interrupted and then restored. IRI occurs in a broad spectrum of clinical settings, including surgery, trauma or sepsis that leads to the functional disturbances of the kidneys. During transplantation, IRI is a risk factor for delayed graft function, which prolongs hospitalization and increases the cost. However, in recent years, ischemic conditioning methods have been discussed as powerful protective techniques to reduce the extent of renal IRI. These novel approaches have shown promising results in animals and research are underway to examine their exact effect in human in the clinical scenarios.

## Different types of conditioning methods

In brief, “ischemic conditioning” is the application of transient episodes of ischemia against a prolonged lethal ischemia. Prior sublethal local ischemic periods induce a state of protection that is called ischemic preconditioning (IPC). This was first demonstrated in a study by Cochrane *et al*. in an animal model of renal IRI (1). However, the use of IPC in the treatment of acute ischemic situation is limited. It therefore became necessary to develop new methods applicable against unpredictable ischemic scenarios. One option is the modification of the reperfusion phase by means of brief renal artery occlusions and reperfusions applied at the onset of renal reperfusion, a phenomenon called ischemic post-conditioning (IPOC).

Several studies have demonstrated that IPOC, intermittent interruptions of blood flow at the onset of reperfusion, can reduce myocardial infarct size by as much as 40% in animals. The renoprotective effect of this technique was reported by many investigations including the study by Liu *et al*. in 2007 (2). Beneficial effects of IPOC have also been observed in humans, for example after cardiac surgery and with acute myocardial infarction. A shortcoming of both preconditioning and post-conditioning, as local or classical conditionings, is that manipulation of the main ischemic organ by invasive techniques may lead to serious, life-threatening complications. Elongation of the operation time may be another negative aspect. Thus, this strategy has its own clinical limitations and is mostly applicable to patients undergoing manual reperfusion.

## Remote conditioning

Another alternative approach is “remote ischemic conditioning” triggered by brief IR of a distant organ. Similar to local conditioning techniques (IPC and IPOC), remote ischemic conditioning can be performed before main organ ischemia (remote ischemic preconditioning, r-IPC) or at the onset of reperfusion (remote ischemic post-conditioning, r-IPOC). Studies have demonstrated renoprotection by remote conditioning in both the animal studies and clinical trials. In a randomized study, Er *et al.* reported that remote ischemic preconditioning before contrast medium use prevents contrast medium-induced acute kidney injury in high-risk patients (3).

Interestingly, this novel method can also be applied during the main organ ischemia. In this regard, when the site of ischemic conditioning is located remotely, the conditioning cycles can be applied anytime during the target organ ischemia, a novel phenomenon known as remote ischemic per-conditioning (r-PEC).

We have recently, applied limb ischemic conditioning at the beginning of renal ischemia, to protect the kidney from the subsequent renal IR injury. This was published as the first report of renoprotective effect of r-PEC in a 2011 edition of Transplant journal (4). We have also shown that r-PEC reduces oxidative stress and down-regulates cyclooxygenase-2 expression in a rat model of ischemia/reperfusion-induced acute kidney injury (5).

In the clinical studies, r-PEC has shown desirable cardio-protective effects when used as an additional treatment in case of valve replacements (6). This method has the advantage of eliminating additional manipulation of the main visceral organ or vessel involved. It can be simply applied using blood pressure cuffs placed around the proximal part of the limb. The essence of this method is that brief, remote ischemic attacks are applied after the beginning of the induction of target organ ischemia, but before the onset of reperfusion. Therefore, it overwhelms the disadvantages of r-IPC and r-IPOC, regarding unpredictable feature of the onset time of both ischemia (for r-IPC) and reperfusion (for r-POC), as well as shortening of the operation time.

The possibility to create resistance against ischemic injury through ‘organ conditioning’ is an area of increasing interest. The remote ischemic conditioning may have an immense effect on the renal patients and in clinical practice in the near future. r-PEC is perhaps the most promising protective strategy among the adaptive surgical techniques.

## Authors’ contributions

All authors wrote the manuscript equally.

## Conflict of interests

The authors declared no competing interests.

## Ethical considerations

Ethical issues (including plagiarism, data fabrication, double publication) have been completely observed by the author.

## Funding/Support

None.
